# Moving towards a unified classification of glioblastomas utilizing artificial intelligence and deep machine learning integration

**DOI:** 10.3389/fonc.2023.1063937

**Published:** 2023-06-23

**Authors:** Ciaran Scott Hill, Anand S. Pandit

**Affiliations:** ^1^ Institute of Neurology, University College London, London, United Kingdom; ^2^ Victor Horsley Department of Neurosurgery, National Hospital for Neurology and Neurosurgery (NHNN), London, United Kingdom

**Keywords:** glioma, glioblastoma, classification, artificial intelligence, machine learning

## Abstract

Glioblastoma a deadly brain cancer that is nearly universally fatal. Accurate prognostication and the successful application of emerging precision medicine in glioblastoma relies upon the resolution and exactitude of classification. We discuss limitations of our current classification systems and their inability to capture the full heterogeneity of the disease. We review the various layers of data that are available to substratify glioblastoma and we discuss how artificial intelligence and machine learning tools provide the opportunity to organize and integrate this data in a nuanced way. In doing so there is the potential to generate clinically relevant disease sub-stratifications, which could help predict neuro-oncological patient outcomes with greater certainty. We discuss limitations of this approach and how these might be overcome. The development of a comprehensive unified classification of glioblastoma would be a major advance in the field. This will require the fusion of advances in understanding glioblastoma biology with technological innovation in data processing and organization.

Gliomas represent the most common primary brain cancer. They have distinct biological features and clinical behavior, and account for nearly 80% of the malignant brain tumors in adults (1, 2). The commonest subtype of glioma is glioblastoma a deadly brain cancer that is nearly universally fatal. Understanding of natural history, accurate prognostication, therapeutic efficacy, and the successful application of emerging precision medicine in glioblastoma relies upon the resolution and exactitude of classification. The WHO classification of Central Nervous System tumors began in 1970 (3). The first edition was largely based on anatomical and histological findings. Many of the major shifts in neuro-oncology and glioblastoma understanding over the intervening years have been represented in the subsequent WHO classification updates and the associated cIMPACT-NOW statements (4). A major conceptual leap was made in 2012 with the recognition of key subclassification of glioblastoma based on IDH mutation status (10.1038/nature10860.). This single mutation cleaved glioblastoma into two major subtypes with differing etiology, therapeutic vulnerability, and prognosis. In 2021 the significance of this stratification became codified by separating glioblastoma (IDH wild type) fully from grade 4 diffuse astrocytoma with IDH mutation (5).

In addition to the formal WHO classification there have been a multitude of differing stratifications of glioblastoma categorizations based on transcriptional profiles. A major development was in 2006 when Phillips et al. published a transcriptional classification of high-grade glioblastoma (6). This was advanced in 2010 when Verhaak et al. used data derived from The Cancer Genome Atlas to sub-stratify into 4 subgroups; proneural, mesenchymal, classical, and neural (7, 8). These were reported to have differing prognosis and treatment vulnerabilities. Further modifications and refinements to transcriptional groups, including single-cell profiling of both the tumor cells and microenvironmental components such as the neuro-immune niche have since been proposed by several groups including Neftel et al (9) and Richards et al (10). Another layer of complexity was added by epigenetic DNA-methylation profiling. This has already had significant clinical impact in supporting diagnosis and risk stratification (11, 12). Single-cell level profiling is not limited to transcriptional RNA profiling, it can also be applied across a range of biological analytic technologies including proteomic analysis - this opens up unparalleled levels of biological data.

Technologies in spatial-omics enable a greater understanding of cellular organizations and interactions within a tissue of interest. This is particularly of interest in cancer biology and can be applied at microscopic and super-resolution levels across the full range of spectral wavelengths and including spectroscopy data (13–16).

However, histology, selective mutations, transcriptional profiles and epigenetic changes do not tell the full story of glioblastoma diversity. One of the major barriers to successful new therapies in glioblastoma is considered the intra- and inter- heterogeneity of the tumors and this extends beyond these molecular sources of variability. In addition to transcriptional and epigenetic variability, anatomical location and structural features – including presence of cysts, degree of necrosis, proliferation indices etc (17), variability in radiomic findings (18), whole genome genetic/mutational characteristics (potentially including variability in extra-chromosomal sites) (19, 20) metabolic and lipidomic (21) and proteomics (22) can all be used to codify glioblastoma.

Integrating all these variables into a unified classification which reflects the diversity of glioblastoma states and in a clinically relevant manner, represents a daunting task (see **Figure 1**). Without this nuanced lamination we continue to risk masking the efficacy of new therapies by disease heterogeneity leading to variability of response. Likewise, our ability to accurately provide disease prognostication will remain limited.

**Figure 1:**
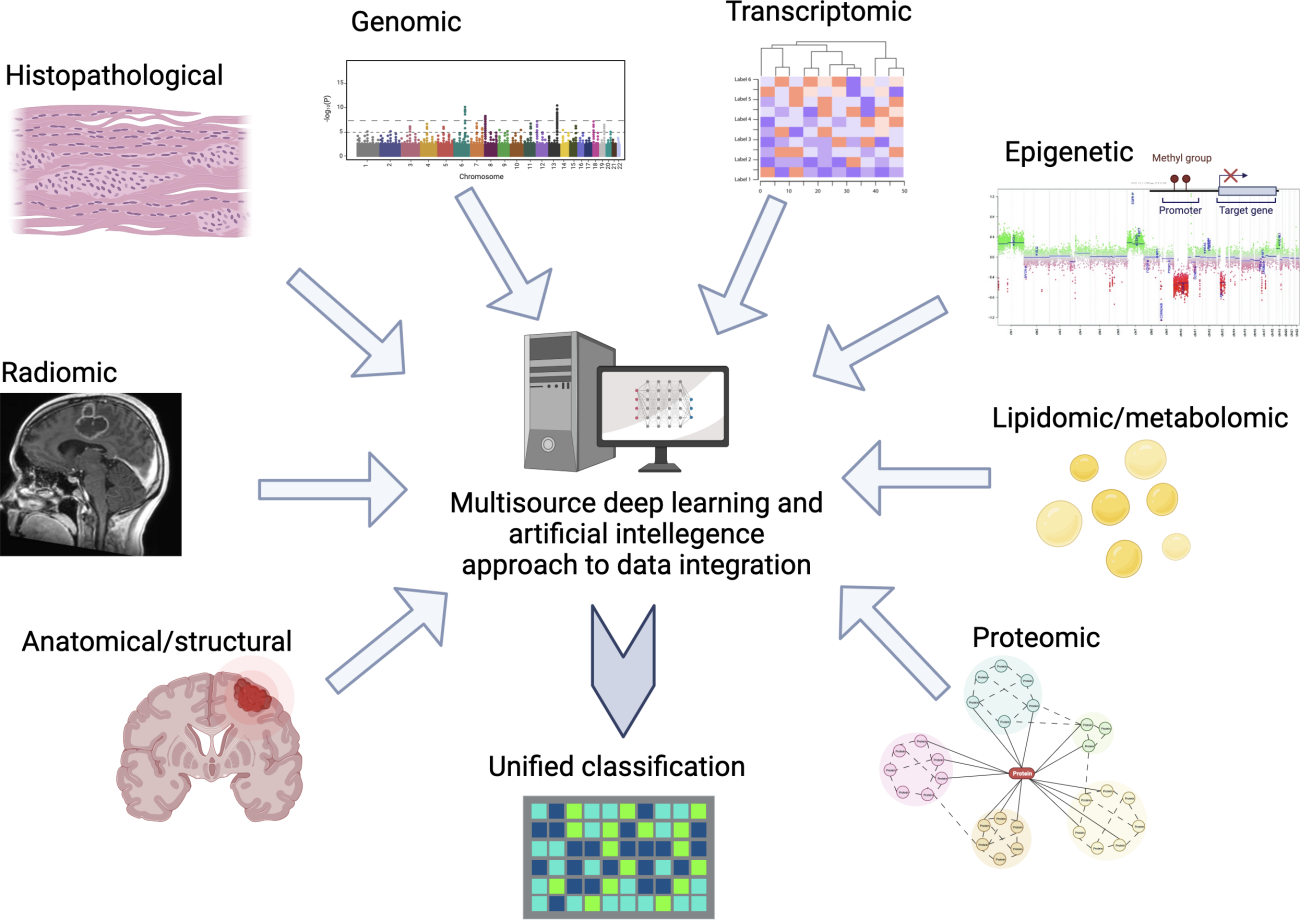
Example of multisource data input and integration for deep learning guided classification of glioblastoma.

Artificial intelligence (AI) tools provide the opportunity to organize and integrate these factors to generate clinically relevant disease sub-stratifications, which would help predict neuro-oncological patient outcomes with greater certainty. With enough data, DL (deep learning) methods based on neural networks have emerged as a leading approach for capturing highly informative features in oncology datasets. Using these tools, rapid progress has been made in each of the modalities described above. However, unanswered questions remain about how multimodal data can be integrated and a unified classification model be built.

A key requirement of multimodal integration is that each data source complements the others, enhancing information content beyond the scope of any single modality. For example, radiological data on macroscopic tumor morphology, as well as molecular and histological data, describe disease from different perspectives and scales. Each data source in a unified model should be at least partially orthogonal to the next.

While multimodal patient stratification methods have been developed for cancer patients in general, these mainly rely on *multi-omic* (multi-dimensional genomic data) in the absence of radiological or clinical information (23), and there currently exist few examples which utilize multimodal strategies for glioma patients specifically. Among these, there have been single-center studies which stratify glioma patients using multiparametric MRI, molecular and transcriptome information using kernel based learning (24), and deep learning approaches to predict survival which integrate both histological and genomic (but not radiological) information based on gliomas from The Cancer Genome Atlas (25, 26). These studies suggest that multimodal integration improves patient stratification and outcome prediction over unimodal methods.

Given its purported success, what is limiting this type of work? Many major limitations are simply related to the lack of availability of large, annotated datasets with multimodal information streams, which are sufficiently rich and class-balanced that the breadth of glioma heterogeneity can be encompassed. Other limitations pertain to how individual data modalities should be fused. It is unclear whether raw data should be concatenated from the start and used to train a single model. Or, alternatively, a composite model should be built from learned features, that are each derived from multiple single modality models (27).

In this regard, novel dimension-reduction and clustering methods (28), alongside other techniques which appropriately weigh will help in leveraging the vast amount of collected multimodal parameters for each patient and help prevent overfitting (29, 30). Finally, interpretation of deep learning models is notoriously difficult, and if clinicians want to understand how a unified model relates to the disease process, methods to make such models explainable are urgently needed.

Only by developing a comprehensive unified classification of glioblastoma can we optimize our prognostication and maximize the chance of precision therapies being successful. A system that allows integration of ever-increasing complexity and nuance will allow flexibility and adaption to new discoveries and therapies. Given the multiple layers of data involved in glioblastoma biology and their deep complexity and inter-related influence the consolidation and organization into a utilizable structure will require novel approaches. The application of artificial intelligence and deep machine learning in oncology is expanding at an explosive rate with numerous potential applications (31, 32). These technologies will be instrumental in achieving this final goal of a single unified classification of glioblastoma heterogeneity.

## Data availability statement

The original contributions presented in the study are included in the article/supplementary material. Further inquiries can be directed to the corresponding author.

## Author contributions

CH conceived the manuscript. CH and AP co-wrote and reviewed the manuscript. All authors contributed to the article and approved the submitted version.
